# Prize Medals Granted by the Royal Society

**Published:** 1837-01

**Authors:** 


### PRIZE MEDALS GRANTED BY THE ROYAL SOCIETY.

Two medals have been this year granted for physiological papers published in the Society's Transactions during the last three years: THE ROYAL GOLD MEDAL, to George Newport, Esq.; THE COPLEY MEDAL, to Francis Kiernan, Esq., F.R.S., both members of the Royal College of Surgeons.

The two papers of Mr. Newport, for which the Royal Gold Medal has been awarded, are published in the Philosophical Transactions for the years 1834 and 1836. There is also a previous paper, not included within the period of award. The first paper consists of a description of the nervous system of the *Sphinx Ligustri*, or Privet Moth, during the larva and the earlier stages of its pupa state. The second paper is a continuation of the first, and contains a description of the nervous system of the same insect during the remaining part of its pupa state, and also during its perfect state, with the manner in which several ganglia which exist in the larva separately, are aggregated together to form the great thoracic and cephalic masses, and for the development of the optic nerves. The author dwells particularly upon certain peculiar nerves in this and other lepidopterous insects, which have long been well known to anatomists, but which are now described more minutely than has hitherto been done, and which, from their being distributed especially to the breathing orifices, tracheæ, and muscles, along the sides of the insect, he designates *transverse* or *respiratory* nerves. He also minutely describes the structure of the two nervous cords which are analogous to the spinal cord of man and other vertebrata; and shows that, in insects and crustacea, and by analogy in other invertebrata, these two nervous cords are each composed of two tracts, as in the human body; one of these, that which lies nearest the external surface of the body, possesses ganglia, and is believed to be the sensitive; while the second, which is nearest the viscera, like the motor tract in man, is without ganglia, closely approximated to the first, and is only clearly distinguished when it passes over the ganglia.* It is upon and above these double cords that the respiratory nerves are situated, namely, nearest to the viscera. The author describes also, in the same paper, a series of corresponding changes in the nervous system in the common nettle butterfly, similar to those described by Herold in the cabbage butterfly.

The other paper by the same author, which has just appeared in the Society's Transactions for 1836, second part, is on the Respiration of Insects. It consists of a minute description of all the parts concerned in respiration, the tracheæ, spiracles, muscles, and nerves distributed to them; the manner in which respiration is performed in insects, the condition of respiration at particular periods and states of the insect; and the duration of life in different media. In this paper many of the facts are in confirmation of the views of Dr. Marshall Hall, respecting Hybernation, of Edwards and of Treviranus. The three papers are all illustrated by plates, and do honour not only to the author, but to British physiology. We doubt not but Mr. Newport is destined to extend greatly the boundaries of our knowledge of the anatomy and physiology of the Invertebrata, and, through these, to advance our knowledge of the physiology of man.

The paper of Mr. Kiernan, for which he has received the Copley Medal, is that containing his researches into the minute anatomy of the liver, and which has been universally admitted to constitute one of the most important additions which scientific anatomy has received for many years. Mr. Kiernan's investigations and discoveries have been laid so fully before the profession, in the various medical journals, that we deem it unnecessary to give any account of them in this place.

